# Performance Analysis of Orthogonal Multiplexing Techniques for PLC Systems with Low Cyclic Prefix Length and Symbol Timing Offset

**DOI:** 10.3390/s23094363

**Published:** 2023-04-28

**Authors:** Túlio Fernandes Moreira, Ândrei Camponogara, Sobia Baig, Moisés Vidal Ribeiro

**Affiliations:** 1 Department of Electrical Engineering, Federal University of Juiz de Fora, Juiz de Fora 36036-900, Brazil; 2 Department of Electrical Engineering, Federal University of Paraná, Curitiba 80060-000, Brazil; 3 Energy Research Center, Electrical and Computer Engineering Department, COMSATS University Islamabad, Lahore Campus, Islamabad 54000, Pakistan

**Keywords:** power line communication, multiplexing modulations, interference, cyclic prefix, symbol timing offset

## Abstract

This paper investigates the degradation caused by interference resulting from cyclic prefix violation and symbol timing offset in narrowband power line communication systems. In this sense, it presents a unified formulation from which Hermitian symmetric orthogonal frequency division multiplexing (HS-OFDM), orthogonal chirp division multiplexing (OCDM), single-carrier cyclic prefix (SCCP), and orthogonal time–frequency division multiplexing (OTFDM) can be easily derived. The paper then provides closed-form expressions for quantifying the aforementioned interference in the presence of a frequency domain equalizer. The numerical analyses exhibit the performances of these schemes under various data communication conditions, such as the availability of channel state information, the presence or absence of interference, modeling of additive noise as a white or colored Gaussian random process, frequency domain equalizer type, and the use of bit and power allocation techniques. The closed-form expressions and performance analyses regarding achievable data rate and bit error probability provide guidance for dealing with distinct constraints in narrowband power line communication (PLC) systems using the HS-OFDM, OCDM, SCCP, or OTFDM scheme. Lastly, the unified formulation and results obtained motivate the design of multi-scheme transceivers.

## 1. Introduction

Even though electric power systems present many impairments for data communication purposes, the ever-increasing demand for connectivity has brought the attention of both industrial and academic spheres back to this challenging data communication medium. Indeed, modern society’s necessity for connectivity has rushed the development of telecommunication infrastructure and technology to enable Industry 4.0/5.0, the Internet of Things, smart grids, and smart cities [1,2,3]. To overcome the impairments imposed by electric power systems for transmitting information-carrying signals, frequency selectivity effects and the presence of high-power impulsive noise, remarkable advances in the link and Physical (PHY) layers have been introduced in the past three decades [4,5].

Regarding the PHY layer, it is a well-established fact that the multicarrier scheme known as Hermitian symmetric orthogonal frequency division multiplexing (HS-OFDM), which is the orthogonal frequency division multiplexing (OFDM) version for baseband data communication, is widely applied to deal with the aforementioned power line communication (PLC) impairments. It divides the frequency bandwidth into several subbands (i.e., orthogonal subchannels), which are useful for overcoming the frequency selectivity of PLC channels, and consequently maximizing the data rate or minimizing bit error rate (BER) [6,7,8,9,10,11]. It can even be used with a blanker to mitigate impulsive noise disturbances [12,13]. Moreover, the single carrier cyclic prefix (SCCP) scheme was considered as an alternative to the OFDM in [14], showing competitive BER performance in comparison to the OFDM when the normalized signal-to-noise ratio (nSNR) in the frequency domain is not frequency selective. The orthogonal division multiplexing (OCDM) scheme, which combines the orthogonality principle with the chirp spread spectrum (CSS) to yield a multichirp scheme [15], was first applied to PLC systems in [16]. The attractiveness of the OCDM scheme relies on the use of the discrete Fresnel transform (DFnT) implemented with the discrete Fourier transform (DFT). Recent contributions have demonstrated the usefulness of OCDM for optical, underwater acoustics, and sensing applications [17,18,19,20]. Moreover, Colen et al. [21] introduced the orthogonal time–frequency division multiplexing (OTFDM) scheme to reduce the computational complexity of bit and power allocation. The OTFDM is based on the discrete orthogonal Stockwell transform (DOST) [22], which is mostly used in digital image processing with its fast transform application [23,24]. An analysis of the mathematical descriptions of these schemes shows the existence of relationships among them, which can be exploited to design multi-scheme transceivers for dealing with distinct sets of constraints in PLC systems.

Moreover, the literature shows that the HS-OFDM scheme for PLC systems has been widely and deeply investigated. Consequently, it has been introduced in several standards and protocols; however, we cannot say the same for the other aforementioned schemes. Considering the OCDM scheme, the literature shows investigations considering the carrier frequency offset and narrowband interference in wireless systems [25]. Moreover, the authors in [16] compared the HS-OFDM scheme, SCCP, and OCDM sensitivity to the cyclic prefix (CP) length violation based on the Monte Carlo Simulation. Considering the OTFDM schem, [21] introduced and applied it to reduce computational complexity during the resource allocation process while [26] provided an initial discussion about the CP length violation and the symbol timing offset (STO). Unfortunately, the available literature does not show how these schemes, under the presence of the CP length violation, behave when PLC channels are corrupted by white or colored additive noises, and different techniques are applied to perform resource allocation and frequency domain equalization.

Aiming to deal with the aforementioned issues, this paper focuses on the performance comparison among HS-OFDM, OCDM, SCCP, and OTFDM schemes in the presence of the CP length violation and the STO in data communication performed by baseband PLC systems, in which the media is characterized by frequency-selective channel frequency response (CFR). The main contributions of this study are as follows. We present:

- A presentation of a unified formulation from which the HS-OFDM scheme, OCDM, SCCP, and OTFDM schemes are derived for baseband systems, as well as a deduction of closed-form expressions for the estimated signal, signal–to–interference–plus–noise ratio (SINR), and SINR upper-bound when frequency domain equalizers (i.e., complete zero–forcing (C-ZF), modified zero–forcing (M-ZF), and single–tap zero–forcing (ST-ZF) [27]) are considered.
- Numerical analyses to compare closed-form expressions with the Monte Carlo simulation and different types of frequency domain equalizers; we show that the STO might benefit data communication systems when the CP length violation occurs.
- Performance analyses comparing HS-OFDM, OCDM, SCCP, and OTFDM in terms of the achievable data rate, which considers uniform power allocation (UA) and optimal power allocation (OA), and bit error probability (BEP) with adaptive modulation when the frequency domain equalizer is used to deal with linear time–invariant (LTI), PLC, and channel impulse response (CIR), which is frequency selective and corrupted by the presence of additive noise modeled as a white or colored random process.

Our major findings are:

- The unified formulation allows us to derive HS-OFDM, OCDM, SCCP, and OTFDM schemes easily. Consequently, we advocate that it can support future designs of transceivers based on multi-schemes. Moreover, the closed-form expressions can correctly characterize the performance of these schemes. Consequently, it is unnecessary to perform Monte Carlo simulations.
- For all schemes, the STO might benefit the system performance when the CP length violation occurs. In other words, small values of STO can relieve the interference caused by the CP length violation. In the scenario without the CP length violation, we show the transmission block will not be degraded by the interference caused by the channel spreading if the sum of the CP length with the STO is bigger than the length of PLC CIR minus one.
- Similar behavior is observed in the narrowband PLC systems using the aforementioned schemes when the C-ZF scheme, M-ZF, and ST-ZF are applied. For instance, the C-ZF scheme and ST-ZF always attain the best and the worst performances for all schemes, respectively.
- Regarding the achievable data rate analysis, we see that without interference (i.e., no CP length violation and no STO), the HS-OFDM scheme always attains the highest achievable data rate, followed by OTFDM. In the sequel, we have SCCP and OCDM, which yield the same achievable data rate. On the other hand, under the interference’s presence, the HS-OFDM scheme attains the highest achievable data rate when the total transmission power belongs to a certain range of values. Above this range of values, OTFDM attains the highest achievable data rate. Moreover, HS-OFDM offers the lowest achievable data rate upper bound while SCCP attains the highest.
- The BEP analyses for all scenarios with interference show that OCDM attains the lowest bit error probability. It is followed by HS-OFDM, SCCP, and OTFDM, in this order. Note that SCCP and OTFDM attain similar BEP performances and have higher lower bounds. On the other hand, in the absence of interference, the best BEP performances are attained by OCDM and SCCP, followed by OTFDM, with HS-OFDM being the worst.

The rest of this paper is organized as follows: Section 2 details the system model; Section 3 focuses on the transmitter and receiver general mathematical formulation, which includes the CP length violation and the STO; it also discusses the types of interference that corrupt the data communication system. Section 4 shows how to derive the OCDM scheme, SCCP, and OTFDM schemes from the unified formulation and it also discusses their similarities and computational complexities; Section 5 focuses on the performance comparisons among HS-OFDM, OCDM, SCCP, and OTFDM schemes in terms of data rate and BEP; and, finally, Section 6 outlines our concluding remarks.

## 2. System Model

**Figure 1:**
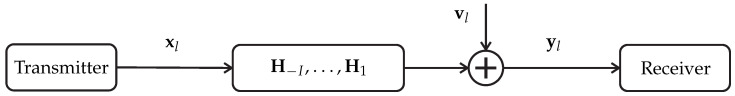
Block diagram of a PLC communication system.

A block diagram for the baseband system model in the discrete-time domain is illustrated in Figure 1. It comprises a generic transmitter, a filtered additive channel model, and a generic receiver. It considers a vector representation for modeling the data communication through an LTI PLC channel, in which the output is corrupted by the presence of an additive noise modeled by a zero-mean and wide–sense stationary (WSS) random process. The time-invariant assumption is made because the time interval for transmitting a $\overset{˜}{N}$-length symbol block is much shorter than the coherence time of PLC channels. The vector $h={\left[h\left[0\right],\ldots ,h\left[L_{h}-1\right]\right]}^{T}$, where ${\left(\cdot \right)}^{T}$ is the transpose operator and h[n] is the *n*th sample of the CIR, denotes a vector representation of an $L_{h}$-length PLC channel CIR. Note that the CFR of such a PLC channel is also supposed to be frequency-selective.

To build the $l^{\mathrm{th}}$$\overset{˜}{N}$-length transmitted block, we assume that an $L_{CP}$-length CP is pre-appended to the 2N-length sequence of symbols, generating $x_{l}\in R^{\overset{˜}{N}\times 1}$, where $\overset{˜}{N}=2N+L_{\mathrm{CP}}$ and $L_{\mathrm{CP}}\leq L_{h}-1$. Further, the vector representation of the CFR in the discrete frequency domain is given by $H=\sqrt{2N}F{\left[h^{T}0_{(2N-L_{h})\times 1}^{T}\right]}^{T}\in R^{2N\times 1}$, where $F\in C^{2N\times 2N}$ is the normalized DFT matrix with elements $F\left[n,m\right]=\frac{1}{\sqrt{2N}}e^{-j\frac{2\pi }{2N}nm}$, (m,n)=0,1,…,2N−1, and $0_{C\times D}$ is a (C×D)-size matrix with entries equal to zero.

**Figure 2:**
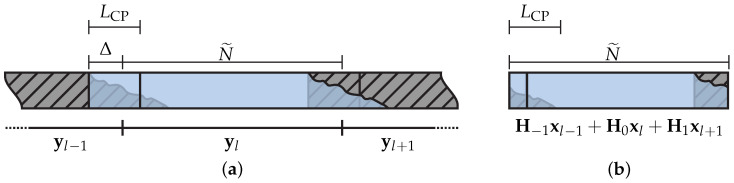
Representation of (1) with I=1 and $v_{l}=0_{\overset{˜}{N}\times 1}$. (**a**) A sequence of three consecutive $\overset{˜}{N}$—length receiving blocks. (**b**) The *l*th receiving block or $y_{l}$.

Now, we assume a lack of perfect synchronization at the receiver and, consequently, the presence of STO, which is quantified by the variable Δ∈Z. Considering the presence of inter–symbol interference (ISI), STO, and the additive noise, the vector representation of the *l*th $\overset{˜}{N}$-length received block can be expressed as [28]

(1) $$y_{l}=\sum_{i=-1}^{I}H_{-i}x_{l-i}+v_{l},$$

where $x_{l-i}\in R^{\overset{˜}{N}\times 1}$ represents the (l−i)th transmitted block, −1≤i≤I, with $I=\lceil L_{h}/\overset{˜}{N}\rceil$ being the number of transmitted blocks that may cause interference because of the channel spreading, and ⌈z⌉=min{a∈Z|a≥z} being the ceiling function; $v_{l}\in R^{\overset{˜}{N}\times 1}$ is the vector representation of the additive noise. Moreover, $x_{l-i}$ and $v_{l}$ are independent and zero-mean WSS random vectors, and $H_{-i}\in R^{\overset{˜}{N}\times \overset{˜}{N}}$ is the channel CIR convolution matrix with entries

(2) $$\begin{array}{c} H_{-i}\left[n,m\right]=\left\{\begin{array}{cc} 0, & \mu +n-m<0\\ h[\mu +n-m], & 0\leq \mu +n-m\leq L_{h}-1\\ 0, & L_{h}-1<\mu +n-m, \end{array}\right. \end{array}$$

where $\mu =i\overset{˜}{N}+\Delta$ is the first sample of the (l−i)th received block with reference to the *l*th received block. Figure 2 shows a visual representation of (1) with $\overset{˜}{N}\gg L_{\mathrm{CP}}$ and $v_{l}=0_{\overset{˜}{N}\times 1}$.

In the following section, we provide a detailed general formulation from which four multiplexing techniques are derived. These four schemes have distinct characteristics for dealing with typical operating conditions, such as complete or incomplete channel state information (CSI), cyclic prefix violations, frequency selectivity of the magnitude of CFR, power spectral density (PSD) of the additive noise, and nSNR.

## 3. General Formulation

**Figure 3:**
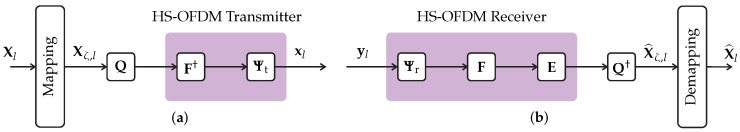
Block diagrams of the generic transmitter and receiver. (**a**) Generic transmitter; (**b**) generic receiver.

The multiplexing modulation schemes considered in this paper are HS-OFDM, OCDM, SCCP, and OTFDM, which are configured to operate in the baseband. Note that HS-OFDM refers to the baseband version of the OFDM. Figure 3 shows a block diagram for a generic transmitter and receiver, which covers these four schemes and applies to the transmitter and receiver used to transmit data through the PLC channel model detailed in Section 2. Concise descriptions of both of them and the interference are presented in the following subsections.

### 3.1. Generic Transmitter

The block diagram of the generic transmitter is shown in Figure 3a. The *N*-length block of the points belonging to *M*-ary constellations is represented by the symbol vector $X_{l}\in C^{N\times 1}$ in the discrete frequency domain. In the sequel, $X_{l}$ is properly mapped to be used in a baseband data communication system, such as the PLC. The mapping process results in the mapped vector $X_{\zeta ,l}\in C^{2N\times 1}$ being in the discrete frequency domain. The details of this mapping process can be found in [8] for the HS-OFDM scheme, [21] for the OTFDM schem, and [16] for both the OCDM scheme and SCCP.

Then, the mapped vector is processed through a precoding and unitary matrix, Q, such that $Q^{†}Q=I_{2N}$, where $I_{C}$ is an identity matrix of size *C* and ${\left(\cdot \right)}^{†}$ is the conjugate transpose operator. This precoding matrix defines which one of the multiplexing modulation schemes is being used, following

(3) $$Q=\left\{\begin{array}{cc} I_{2N} & \mathrm{for}\;\mathrm{the}\;\text{HS-OFDM}\;\mathrm{scheme}\\ \Gamma_{\mathrm{HS}}^{†}F & \mathrm{for}\;\mathrm{the}\;\mathrm{OCDM}\;\mathrm{scheme}\\ F & \mathrm{for}\;\mathrm{the}\;\mathrm{SCCP}\;\mathrm{scheme}\\ {\bar{D}}^{†} & \mathrm{for}\;\mathrm{the}\;\mathrm{OTFDM}\;\mathrm{schem} \end{array}\right..$$

The final step in the generic transmitter comprises the inverse discrete Fourier transform (IDFT) matrix, $F^{†}$, and the cyclic prefix matrix, which is equal to

(4) $$\Psi_{t}=\left[\begin{array}{c} 0_{L_{\mathrm{CP}}\times \left(2N-L_{\mathrm{CP}}\right)}\;\;\;\;I_{L_{\mathrm{CP}}}\\ I_{2N} \end{array}\right].$$

Therefore, we can define a generic transmission matrix as

(5) $$T≜\Psi_{t}F^{†}Q,$$

and, consequently, the vector representation of the *l*th transmitted block is given by

(6) $$\begin{array}{cc} x_{l} & =TX_{\zeta ,l}. \end{array}$$

### 3.2. Generic Receiver

The block diagram for the generic receiver is shown in Figure 3b. First, the received block at the receiver input, given by (1) with $x_{l-i}$, and equal to (6), is submitted to the removal of the CP, which is accomplished with the matrix $\Psi_{r}=\left[0_{2N\times L_{\mathrm{CP}}}\;I_{2N}\right]$. After that, the normalized DFT matrix, F, and the frequency domain equalizer E apply. In the sequel, the estimated 2N-length symbol block is obtained after using the inverse of the precoding matrix $Q^{†}$. Consequently, the generic receiver matrix is defined as

(7) $$R≜Q^{†}\mathrm{EF}\Psi_{r}.$$

Depending on the type of frequency domain equalizer, distinct levels of interference mitigation can be accomplished. According to [27], frequency domain equalizers offering three distinct levels of interference mitigation are given by

(8) $$E=\left\{\begin{array}{cc} B_{0}^{†}{\left(\sum_{i=-1}^{I}B_{i}^{†}B_{i}\right)}^{-1}, & \mathrm{for}\;\text{C-ZF}\\ {\left(B_{0}^{†}B_{0}\right)}^{-1}B_{0}^{†}, & \mathrm{for}\;\text{M-ZF}\\ \Lambda_{B_{0}}^{-1}, & \mathrm{for}\;\text{ST-ZF} \end{array}\right.,$$

where $\Lambda_{B_{0}}$ is obtained assuming that a full matrix Z is decomposed into its diagonal ($\Lambda_{Z}=\mathrm{diag}\left\{Z\right\}$) and off-diagonal components (${\bar{\Lambda }}_{Z}=Z-\Lambda_{Z}$), and $B_{i}=F\Psi_{r}H_{-i}\Psi_{t}F^{†}$. Note that the C-ZF scheme attains the best interference mitigation and demands the highest processing power. In contrast, ST-ZF offers the worst interference mitigation and demands the lowest processing power. Moreover, M-ZF achieves a performance (and demands a processing power) between C-ZF and ST-ZF.

Applying (7) in (1) while considering (6) results in the estimated mapped vector, which corresponds to an estimation of $X_{\zeta ,l}$, given by

(9) X^ζ,l=∑i=−1IRH−iTXζ,l−i+Rvl=RH0TXζ,l+∑i=−1i≠0IRH−iTXζ,l−1+Q†EVl=ΛA0Xζ,l︸attenuatedvector+Λ¯A0Xζ,l+∑i=−1i≠0IAiXζ,l−1︸interferencevector+GVl︸enhanced-noise.

where $A_{i}={\mathrm{RH}}_{-i}T$, $G=Q^{†}E$ is the noise-enhancement matrix, $V_{l}=F\Psi_{r}v_{l}$ is the noise in the frequency domain. The final step of the receiver is the demapping process.

**Figure 4:**
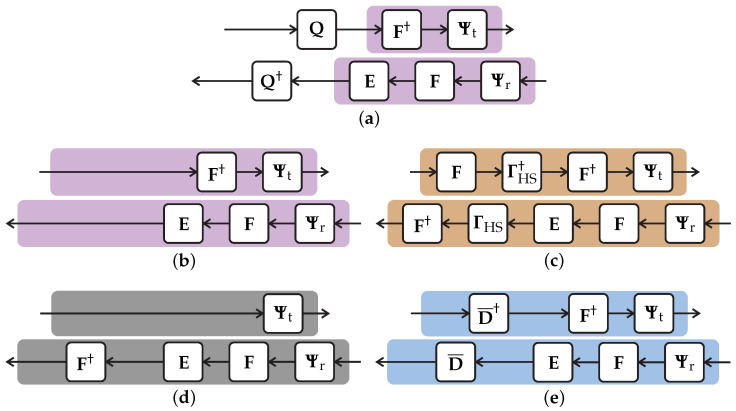
The mathematical formulation of the generic transmitter and receiver for deriving the four digital modulation schemes. (**a**) The generic transmitter and receiver; (**b**) $Q=I_{2N}\to$ HS-OFDM; (**c**) $Q=\Gamma_{\mathrm{HS}}^{†}F\to$ OCDM; (**d**) $Q=I_{2N}\to$ SCCP; (**e**) $Q={\bar{D}}^{†}\to$ OTFDM.

Based on mathematical descriptions of the generic transmitter and receiver, Figure 4 shows how to derive the transmitter and receiver of HS-OFDM, OCDM, SCCP, and OTFDM from the generic transmitter and receiver. In other words, the detailed mathematical formulation shows that the similar compositions of these four data communication schemes opens up the opportunity for a future compact and unified implementation (i.e., low hardware resources) in a PLC transceiver, which can be dynamically switched to deal with specific characteristics of a data communication media, sensed by the PLC system.

### 3.3. Interference in the Data Communication System

If the data communication system is operating with $L_{\mathrm{CP}}<L_{h}-1$ and Δ≠0, two types of interference may be present in the data communication system. The first is the ISI caused by the 2N-length blocks symbols $X_{\zeta ,l-i},i=-1,1,\ldots ,I$, to the desired 2N-length symbol block, and $\Lambda_{A_{i}},i=-1,1,\ldots ,I$ contributes to this interference. The second interference varies with the scheme in use; it is called inter–carrier interference (ICI) for the HS-OFDM scheme, inter–chirp interference (ICpI) for the OCDM scheme, inter–slot interference (IStI) for the SCCP scheme, and inter–tile interference (ITI) for the OTFDM scheme. This second type of interference is caused by other subcarriers (HS-OFDM), subchirps (OCDM), subslots (SCCP), and subtiles (OTFDM) from the (l−i)th 2N-length blocks, i=−1,0,…,I. The matrices ${\bar{\Lambda }}_{A_{i}}$, i=−1,0,…,I comprise this second type of interference.

Moreover, it is easy to see that from (2), a value of Δ=0 results in $H_{1}=0_{\overset{˜}{N}\times \overset{˜}{N}}$, and, as a consequence, in $A_{-1}=0_{2N\times 2N}$ regardless of the $L_{\mathrm{CP}}$ values. Moreover, regarding the channel spreading influence of other 2N-length symbol blocks into the *l*th 2N-length symbol block, the use of $L_{\mathrm{CP}}\geq L_{h}-1$ deals with this issue, i.e., $A_{i}=0_{2N\times 2N}$, i=1,…,I. However, values of Δ>0 also decrease the channel spreading in the target 2N-length symbol block, as shown in (2), which leads to $A_{i}=0_{2N\times 2N}$, i=1,…,I if $L_{\mathrm{CP}}+\Delta \geq L_{h}-1$ as Theorem 1 dictates.

**Theorem** **1.** *Let ${\underline{\bar{H}}}_{-i}=\Psi_{r}H_{-i}\Psi_{t}\in R^{2N\times 2N}$ be the so-called cropped channel CIR convolution matrix, with entries given by*

(10) $${\underline{\bar{H}}}_{-i}\left[n,m\right]=\left\{\begin{array}{cc} 0, & \mu +n-m<0\\ h[\mu +n-m], & 0\leq \mu +n-m\leq L_{h}-1\\ 0, & L_{h}-1<\mu +n-m, \end{array}\right.$$

*with n,m∈[0,2N−1]. If $\Delta \geq L_{h}-1-L_{\mathrm{CP}}$, then ${\underline{\bar{H}}}_{-i}=0_{2N\times 2N}$ for i=1,…,I.*

**Proof.**  Suppose that $\Delta \geq L_{h}-1-L_{\mathrm{CP}}$ and (n−m)∈[−2N+1,2N−1] with $N\in N^{*}$. To show that ${\underline{\bar{H}}}_{-i}=0_{2N\times 2N}$, i=1,…,I, we need to prove that all of its entries are equal to 0. Meaning that the intersection of the interval $A=\{\left(n-m\right)\in Z\;|\;\mu \leq n-m\leq L_{h}-1-\mu \}$ and interval B={(n−m)∈Z|−2N+1≤n−m≤2N−1} must be empty for i=1,…,I and $\Delta \geq L_{h}-1-L_{\mathrm{CP}}$. We can prove that A∩B=∅ by proving that A∩B≠∅ is false. Therefore, we expand A, so that

(11) $$\begin{array}{cc} A & =\{n-m\in Z\;|\;-i\left(2N+L_{\mathrm{CP}}\right)-\Delta \leq n-m\leq L_{h}-1-i\left(2N+L_{\mathrm{CP}}\right)-\Delta \}. \end{array}$$

Hence, for A∩B≠∅ to be true, at least one extremity of B needs to be inside the interval A. Applying the inferior extremity of B first, we have that

(12) $$-i(2N+L_{\mathrm{CP}})-\Delta \leq -2N+1$$

and

(13) $$-2N+1\leq L_{h}-1-i\left(2N+L_{\mathrm{CP}}\right)-\Delta .$$

Therefore, (12) is a correct statement if $\Delta \geq -\left(i-1\right)2N-iL_{\mathrm{CP}}-1$ and since we already established that $\Delta \geq L_{h}-1-L_{\mathrm{CP}}$, then (12) is true. The second inequation is reduced to

(14) $$\Delta \leq -\left(i-1\right)2N-2+L_{h}-iL_{\mathrm{CP}}.$$

and combining it with $\Delta \geq L_{h}-1-L_{\mathrm{CP}}$, we have

(15) $$\begin{array}{cc} L_{h}-1-L_{\mathrm{CP}} & \leq -\left(i-1\right)2N-2+L_{h}-iL_{\mathrm{CP}}\\ 0 & \leq -\left(i-1\right)\left(2N+L_{\mathrm{CP}}\right)-1, \end{array}$$

which is a false statement; hence, (13) is also false. Since −2N+1≤2N−1, we can also state that 2N−1 is not inside the interval A, meaning that A∩B=∅ □

In conclusion, combining $L_{\mathrm{CP}}\geq L_{h}-1$ and Δ=0 results in $A_{0}=I_{2N}$; consequently, (9) reduces to an estimated mapped vector given by

(16) X^ζ,l=Xζ,l+GVl.

Note that (16) is interference-free; however, the frequency domain equalizer may enhance the additive noise.

### 3.4. Signal-to-Interference-Plus-Noise Ratio (SINR)

From (9), it is easy to obtain SINR since the attenuated mapped vector is separated from the interference and additive noise. Indeed, we have that $\Lambda_{A_{0}}X_{\zeta ,l}$ is the attenuated mapped vector, ${\bar{\Lambda }}_{A_{0}}X_{\zeta ,l}+\sum_{\underset{i\neq 0}{i=-1}}^{I}A_{i}X_{\zeta ,l-1}$ is the interference, and ${\mathrm{GV}}_{l}$ is the additive noise contaminated by the noise-enhancement effect. Therefore, the attenuated mapped vector power matrix is given by

(17) $$\begin{array}{cc} P_{s} & =\frac{1}{2N}\Lambda_{A_{0}}E\left\{X_{\zeta ,l}X_{\zeta ,l}^{†}\right\}\Lambda_{A_{0}}^{†}\\ \; & =\Lambda_{A_{0}}P_{x}\Lambda_{A_{0}}^{†}, \end{array}$$

where $P_{x}=E\left\{X_{\zeta ,l}X_{\zeta ,l}^{†}\right\}/2N$ is a diagonal matrix with elements equal to the transmission power. Moreover, the interference and additive noise are independent WSS random processes, and, consequently, the interference-plus-noise power matrix is defined by

(18) $$\begin{array}{c} P_{i+n}≜{\bar{\Lambda }}_{A_{0}}P_{x}{\bar{\Lambda }}_{A_{0}}^{†}+\sum_{\underset{i\neq 0}{i=-1}}^{I}A_{i}P_{x}A_{i}^{†}+GP_{n}G^{†}, \end{array}$$

with $P_{n}=E\left\{V_{l}V_{l}^{†}\right\}/2N$ being a diagonal matrix with entries equal to the noise power. Consequently, the SINR matrix of the 2N-length symbol block is given by

(19) $$\begin{array}{c} \Lambda_{\gamma_{\mathrm{SINR}}}=\frac{\Lambda_{P_{s}}}{\Lambda_{P_{i+n}}}, \end{array}$$

meaning that $\Lambda_{\gamma_{\mathrm{SINR}}}\left[k,k\right]$ is the SINR associated with the $k^{\mathrm{th}}$ subcarrier, subchirp, subslot, or subtile. As shown by expressions (17) through (19), the interference is harmful since it is directly proportional to the transmission power’s strength, meaning that the increase of the transmission power also increases the interference. If the transmission power is extremely high (i.e., $P_{x}\to \infty$), then we can accept that $P_{x}\approx P_{x}I_{2N}$. Consequently, the SINR matrix is expressed as

(20) $$\begin{array}{cc} \Lambda_{\gamma_{\mathrm{SINR},\max}} & =\lim_{P_{x}\to \infty }\frac{\Lambda_{P_{s}}}{\Lambda_{P_{i+n}}}\\ \; & =\frac{\Lambda_{|A_{0}{|}^{2}}}{\Lambda_{|{\bar{\Lambda }}_{A_{0}}{|}^{2}}+\sum_{\underset{i\neq 0}{i=-1}}^{I}\Lambda_{|A_{i}{|}^{2}}}. \end{array}$$

As (20) does not depend on the PSD of the additive noise, we anticipate that the data rate (upper bound) and the BEP (lower bound) will not depend on the PSD of the additive noise. Finally, without interference, $\Lambda_{\gamma_{\mathrm{SINR},\max}}\to \infty$, as expected.

## 4. Data Communication Scheme Derivation

Section 4.1, Section 4.2 and Section 4.3 detail the mathematical formulations for deriving OCDM, SCCP, and OTFDM schemes based on the generic transmitter and receiver detailed in Section 3, while Section 4.4 discusses their nSNR, spectrogram, and computational complexities. The mathematical formulation for deriving OFDM is in [28].

### 4.1. The OCDM Scheme

First, we introduce the chirp band to refer to the dynamic frequency band occupied by a subchirp in the time interval of a $\overset{˜}{N}$-length symbol block.

Let the block diagrams for the OCDM scheme transmitter and receiver be shown in Figure 4c. the OCDM scheme is obtained through the generic formulation by considering $Q=\Gamma_{\mathrm{HS}}^{†}F$, see (3), with the (k,k)th element of $\Gamma_{\mathrm{HS}}$ given by

(21) $$\Gamma_{\mathrm{HS}}\left[k,k\right]=\left\{\begin{array}{cc} e^{-j\frac{\pi }{2N}k^{2}}, & \mathrm{for}\;0\leq k\leq N-1\\ e^{j\frac{\pi }{2N}k^{2}}, & \mathrm{for}\;N-1<k\leq 2N-1 \end{array}\right.,$$

and using the so-called type-IV mapping presented in [16], since it ensures that the OCDM scheme is fit for performing in baseband data communication systems. Therefore, the OCDM scheme transmitter and receiver matrix are equal to

(22) $$T_{\mathrm{OCDM}}=\Psi_{t}F^{†}\Gamma_{\mathrm{HS}}^{†}F$$

and

(23) $$R_{\mathrm{OCDM}}=F^{†}\Gamma_{\mathrm{HS}}\mathrm{EF}\Psi_{r},$$

respectively. With the possession of (22) and (23), we obtain the matrices $A_{i}$, i=−1,…,I and G for the OCDM scheme. Applying (22) and (23) in (19) results in the SINR matrix for the OCDM scheme.

To find the signal–to–noise ratio (SNR) for the OCDM scheme, we must assume that $L_{\mathrm{CP}}\geq L_{h}-1$ and Δ=0. Consequently, the estimated symbol associated with the *k*th subchirp of the 2N-length symbol block, with the vector representation given by (16), is equal to

(24) X^ζ,l[k]=Xζ,l[k]+12N∑i=02N−1ej2π2N(ki−i2)ΛH−1[i,i]Vl[i],

with $E\{V_{l}\left[i\right]\}=0$, $E\left\{V_{l}\left[i\right]V_{l}^{*}\left[i^{\prime }\right]\right\}=E\left\{V_{l}\left[i\right]\right\}E\left\{V_{l}^{*}\left[i^{\prime }\right]\right\}$, $\forall i\neq i^{\prime }$, and $\Lambda_{H}$ standing for the diagonal matrix containing the elements of H. Therefore, the SNR in the *k*th chirp band is expressed as

(25) $$\begin{array}{c} {\underline{\gamma }}_{\mathrm{SNR}}\left[k\right]≜\frac{P_{x}\left[k,k\right]}{\frac{1}{2N}\sum_{i=0}^{2N-1}{\left|\Lambda_{H}^{-1}\left[i,i\right]\right|}^{2}P_{n}\left[i,i\right]}. \end{array}$$

Hence, the nSNR in the *k*th chirp band is given by

(26) $$\begin{array}{cc} {\underline{\gamma }}_{\mathrm{nSNR}}\left[k\right] & ≜{\left(\frac{1}{2N}\sum_{i=0}^{2N-1}\gamma_{\mathrm{nSNR}}^{-1}\left[i\right]\right)}^{-1}, \end{array}$$

where

(27) $$\gamma_{\mathrm{nSNR}}\left[i\right]={\left|\Lambda_{H}\left[i,i\right]\right|}^{2}P_{n}^{-1}\left[i,i\right]$$

is the nSNR associated with the *i*th subcarrier of the well-known HS-OFDM scheme and $P_{n}\left[i,i\right]$ is the element in the position (i,i) of the matrix $P_{n}$. In other words, the nSNR for using the OCDM scheme can be interpreted as a harmonic mean of the nSNR for the HS-OFDM scheme, which raises a few interesting comments. First, (26), being a harmonic mean of nSNR in the HS-OFDM scheme, confirms that a single chirp occupies the whole spectrum during the period of a $\overset{˜}{N}$-length symbol block, while the HS-OFDM scheme states that one subcarrier occupies the same frequency subband during the period of a $\overset{˜}{N}$-length symbol block. Moreover, since all chirp bands have the same nSNR, as shown in (26), the same number of bits are transmitted by all subchirps. In other words, the use of OA and UA results in the same power and bit allocations in all subchirps of an OCDM scheme.

### 4.2. The SCCP Scheme

The block diagram for the SCCP scheme is illustrated in Figure 4d. This scheme is obtained when we adopt Q=F. the SCCP scheme transmitter is given by

(28) $$T_{\mathrm{SCCP}}=\Psi_{t}.$$

Meaning that the transmitter only appends the CP. Consequently, the receiver matrix is expressed as

(29) $$R_{\mathrm{SCCP}}=F^{†}\mathrm{EF}\Psi_{r},$$

which comprises the CP removal matrix and the frequency domain equalization.

Similar to Section 4.1, matrices $A_{i},i=-1,\ldots ,I$, and G are obtained. Moreover, using (28) and (29) results in the estimated mapped vector and the SINR for the SCCP scheme through (9) and (19), respectively.

Moreover, if the data communication system operates without interference, then the *k*th symbol from the 2N-length symbol block, with the vector representation given by (16), is given by

(30) X^ζ,l[k]=Xζ,l[k]+12N∑i=02N−1ej2π2NkiΛH−1[i,i]Vl[i].

Consequently, the SNR associated with the *k*th subslot is given by

(31) $${\underline{\gamma }}_{\mathrm{SNR}}\left[k\right]=\frac{P_{x}\left[k,k\right]}{\frac{1}{2N}\sum_{i=0}^{2N-1}{\left|\Lambda_{H}^{-1}\left[i,i\right]\right|}^{2}P_{n}\left[i,i\right]},$$

and the corresponding nSNR is expressed as

(32) $$\begin{array}{c} {\underline{\gamma }}_{\mathrm{nSNR}}\left[k\right]={\left(\frac{1}{2N}\sum_{i=0}^{2N-1}\gamma_{\mathrm{nSNR}}^{-1}\left[i\right]\right)}^{-1}. \end{array}$$

Straightforwardly, we see that the expressions (31) and (32) are equal to (25) and (26), respectively. This means that SCCP and OCDM will obtain the same performance in terms of BEP and the data rate if both schemes operate free of interference. In the presence of interference, we have to consider the use of the SINR. As the SINR of both schemes differ, SCCP and OCDM attain different performances.

### 4.3. The OTFDM Scheme

First, we introduce tileband to refer to the frequency band occupied by a subtile during a fraction of the period of a $\overset{˜}{N}$-length symbol block.

The OTFDM, proposed in [21], is a data communication scheme that uses the DOST [22] to divide the time–frequency space into orthogonal subtiles bounded by a specific arrangement. This study considers the B-geometry to obtain the subtiles distributions in the time–frequency domain, where B is the geometry order. To obtain the OTFDM scheme from the proposed formulation, we have to consider $Q=\bar{D}$. The (k,i)th element of the matrix $\bar{D}$ is given by

(33) $$\begin{array}{c} \bar{D}\left[k,i\right]=\left\{\begin{array}{cc} \frac{1}{\sqrt{\beta [k]}}e^{j(2\pi \frac{\tau [k]}{\beta [k]}k-\pi \tau \left[k\right])}, & u\leq i\leq U\\ 0, & \mathrm{otherwise} \end{array}\right., \end{array}$$

where u=ν[k]−⌊β[k]/2⌋, U=ν[k]+⌈β[k]/2⌉−1, and ⌊z⌋=max{n∈Z|n≤z} is the floor function. The parameters ν[k], β[k], and τ[k] are elements of the vectors ν, β, and τ, respectively, and are responsible for the creation of each subtile, ensuring that it follows the specific arrangement. In this regard, the *i*th central frequency, subband group size, and position in time are given by ν[k], β[k], and τ[k], respectively. Subtiles that share the same value of ν[k] are called “voice” and an OTFDM scheme using the B-geometry has a voice equal to $\nu =2\log_{2}\left(B\right)+2N/B$ and the subband group related to the *i*th voice is given by $\beta_{\nu }\left[i\right]$, i=0,…,ν−1.

A more detailed explanation of these parameters can be found in [21]. Furthermore, the mapping and demapping process used is the Hermitian symmetric can be found in [8].

Figure 4e illustrates the block diagram for the OTFDM scheme. The transmitter matrix is given by

(34) $$T_{\mathrm{OTFDM}}=\Psi_{t}F^{†}{\bar{D}}^{†},$$

while the receiver matrix is expressed as

(35) $$R_{\mathrm{OTFDM}}=\bar{D}\mathrm{EF}\Psi_{r}.$$

The use of (34) and (35) allow us to obtain matrices $A_{i},i=-1,\ldots ,I$ and G, the estimated mapped vector using (9), and the SINR using (19) for the OTFDM scheme.

For the data communication system that operates free of interference, the estimated symbol associated with the *k*th subtile of the 2N-length symbol block, with the vector representation given by (16), is expressed as

(36) X^ζ,l[k]=Xζ,l[k]+1β[k]∑i=uUej2πτ[k]β[k]ie−jπτ[k]ΛH−1[i,i]Vl[i].

As a result, the SNR in the *k*th tileband is given by

(37) $${\underline{\gamma }}_{\mathrm{SNR}}\left[k\right]=\frac{P_{x}\left[k,k\right]}{\frac{1}{\beta [k]}\sum_{i=u}^{U}{\left|\Lambda_{H}^{-1}\left[i,i\right]\right|}^{2}P_{n}\left[i,i\right]},$$

and, consequently, the corresponding nSNR is equal to

(38) $$\begin{array}{c} {\underline{\gamma }}_{\mathrm{nSNR}}\left[k\right]={\left(\frac{1}{\beta [k]}\sum_{i=u}^{U}\gamma_{\mathrm{nSNR}}^{-1}\left[i\right]\right)}^{-1}. \end{array}$$

Following the comments in Section 4.1 and Section 4.2, we can state that each tileband’s nSNR is a harmonic mean of the subband group of size β[k] centralized at ν[k]. Furthermore, OTFDM can be interpreted as a midterm of the SCCP scheme and HS-OFDM because the choice of the subtile geometry can result in the subband or subslot geometries. It means that the performance of OTFDM is delimited by the performance of HS-OFDM.

### 4.4. General Comments

**Figure 5:**
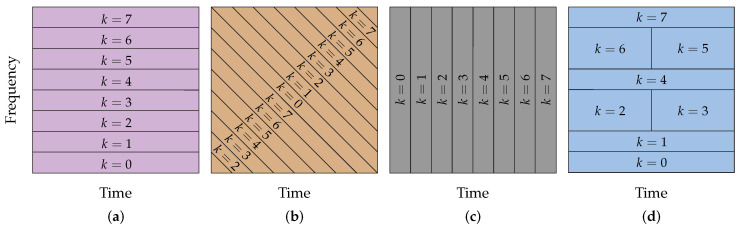
Spectrogram of the four data communication schemes. (**a**) HS-OFDM; (**b**) OCDM; (**c**) SCCP; (**d**) OTFDM.

The four data communication schemes behave differently. Moreover, a valuable illustration is to show the time–frequency domain divisions provided by these schemes when a 2N-length symbol block is considered. In this sense, Figure 5 shows the geometric figures for the time–frequency domain occupation for subcarriers (HS-OFDM), subchirps (OCDM), subslots (SCCP), and subtiles (OTFDM) when 2N=8. We can see that these geometric figures are different and, consequently, the four data communication schemes attain different performances in general.

Moreover, the previous subsections confirm that the generic transmitter and receiver let us see the data communication schemes mentioned above from a unique formulation. Considering interference-free condition (i.e., $L_{\mathrm{CP}}\geq L_{h}-1$, Δ=0), Table 1 shows that nSNR expressions (26), (32), and (38) are very similar to each other since all of them are harmonic means of (27). Moreover, the interference-free condition combined with the flatness of the CFR magnitude and a constant PSD of the additive noise results in the same value as the SNR for the four schemes. We can also see that the SNR for the OTFDM scheme (i.e., (38)) is the harmonic mean of consecutive $\beta_{k}$ subbands, meaning that (38) will be the same fand subtiles that have the same central frequency ν[k], or that are in the same voice. Moreover, an OTFDM scheme using B-geometry was exploited in [21] to reduce the computational complexity of the OA techniques applied to the OTFDM scheme.

Furthermore, a comparison between the nSNR of the four data communication schemes, see Table 1, shows that each subcarrier occupies only a single subband with an nSNR that can be different from other subbands since the CFR of the PLC channel and the PSD of the additive noise may not be flat. Meanwhile, each subchirp of the OCDM scheme occupies a constant bandwidth that linearly shifts in frequency as time evolves. Consequently, the nSNR associated with the OCDM scheme can be interpreted as the harmonic mean of the nSNRs of all subbands. The nSNR for the SCCP scheme is also a harmonic mean of the nSNRs in all subbands since a subslot occupies the whole frequency band. Regarding the OTFDM scheme, we can see that each subtile can occupy pieces of subbands within a given group of subslots. Therefore, the nSNR in a subtile is the harmonic mean of the nSNRs of subbands that constitute a subtile.

Finally, in regard to the computational complexity of each multiplexing modulation scheme, Table 2 summarizes the numbers of basic operations (multiplication and sums) for all transmitters and receivers considered in this paper while considering the fast version of each discrete transform. The HS-OFDM, OCDM, and SCCP computational complexities are found in [16] while OTFDM’s was derived based on [23].

**Figure 6:**
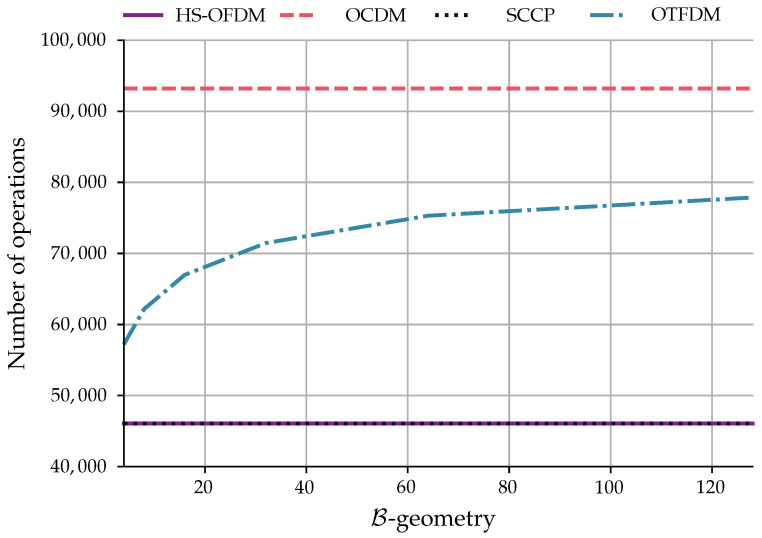
Computational complexity of the schemes when N=256 and $E_{\times }=E_{+}=0$. The abscissa is B-geometry, which shows that we can have different OTFDM schemes with different computational complexities for a value of *N*.

In this context, the frequency domain equalization number of operators is the only distinction between the transmitter and receiver of HS-OFDM, OCDM, and OTFDM, while for the SCCP scheme, they are completely different. Since we are using three different types of equalizers, we define $E_{\times }$ and $E_{+}$ as the multiplication and sum numbers required by the equalizer, respectively, for a generic perspective between the modulation techniques. HS-OFDM and SCCP have the same computational complexity when considering the transmitter and receiver; however, all operations of the SCCP scheme are on the receiver side. Next, the OCDM scheme has more computational complexity than the other two mentioned above. Finally, the OTFDM scheme’s complexity will vary with its B-geometry but will always be lower than the OCDM scheme and higher than the HS-OFDM scheme, as shown in Figure 6.

## 5. Performance Analyses

This section numerically and comparatively analyzes the performance of HS-OFDM, OCDM, SCCP, and OTFDM under the presence of the CP length violation and the STO when a narrowband PLC channel, which is frequency selective, is disturbed by additive noise. The PLC channel is generated following the channel model in [29] and considering the parameters found in [30] (Annex D) with a frequency bandwidth of B=500 kHz starting in f=0 Hz, $L_{h}=30$, and N=256. Based on the values of $L_{h}$ and *N*, we obtain I=1. Consequently, the interference may come only from the (l−1)th and (l+1)th $\overset{˜}{N}$-length symbol blocks. Since our attention is on the frequency selectivity trait of PLC channels, we consider only the background aspect of the PLC noise. Therefore, the additive random process is modeled as a zero-mean and white Gaussian random process, which will be named additive white Gaussian noise (AWGN), or a zero-mean and colored Gaussian random process, which will be named additive colored Gaussian noise (ACGN). The discrete PSD of the AWGN model is equal to $P_{n_{W}}\left[k,k\right]=N_{0}/2$, k=0,1,…,N−1. For the ACGN model, the discrete PSD is expressed as $P_{n_{C}}\left[k,k\right]=\frac{\eta }{2}e^{\left(-v|\Delta fk|\right)}+P_{n_{W}}\left[k,k\right]$, k=0,1,…,N−1, where the constants $v,\eta \in R^{+}$ are equal to $1.2\times 10^{-5}$ and $1.0\times 10^{-15}$, respectively [31], and Δf=B/N.

The parameters used for comparing these data communication schemes are the data rate and BEP. The data rate analysis is based on the UA and OA, through the water-filling algorithm [8], while the BEP analysis relies on the use of UA and adaptive modulation. A discussion about the numerical results obtained with the deduced closed-form expressions and the Monte Carlo simulation, which allows us to check the accuracy of the deduced closed-form expressions, are also detailed.

To perform the data rate analysis based on OA and UA, the assumptions made concerning the channel model (i.e., filtered PLC channel disturbed by the presence of the additive and Gaussian random process) results in the following expression for the achievable data rate [32]:

(39) $$R=\frac{1}{2\left(2N+L_{\mathrm{CP}}\right)T_{s}}\sum_{k=0}^{2N-1}\log_{2}\left(1+\frac{{\underline{\gamma }}_{\mathrm{SINR}}\left[k\right]}{Y}\right),$$

where Y is the gap factor from Shannon’s capacity curve, $T_{s}$ is the sampling time, and ${\underline{\gamma }}_{\mathrm{SINR}}\left[k\right]$ is the *k*th diagonal element of (19) for a given data communication scheme (HS-OFDM, OCDM, SCCP, or OTFDM). Moreover, applying (20) in (39) produces the upper bound for the achievable data rate, which will be denoted by $R^{\mathrm{UB}}$.

The additive noise and interference are considered Gaussian random processes in the frequency domain since the central limit theorem applies in the transformation to obtain the frequency or Fresnel domains. Consequently, the average BEP is expressed as

(40) $$\begin{array}{cc} P_{e} & =\frac{1}{2N}\sum_{k=0}^{2N-1}P_{e}\left[k\right]. \end{array}$$

with $P_{e}\left[k\right]$ being the BEP associated with the $k^{\mathrm{th}}$ subcarrier, subchirp, subslot, or subtle, and given by

(41) $$\begin{array}{cc} P_{e}\left[k\right] & =\frac{4}{\log_{2}\left(M\right)}\left[\left(1-\frac{1}{\sqrt{M}}\right)Q\left(\sqrt{\frac{3{\underline{\gamma }}_{\mathrm{SINR}}\left[k\right]}{M-1}}\right){\left(1-\frac{1}{\sqrt{M}}\right)}^{2}Q{\left(\sqrt{\frac{3{\underline{\gamma }}_{\mathrm{SINR}}\left[k\right]}{M-1}}\right)}^{2}\right], \end{array}$$

where *M* is the constellation order and Q(·) is the Q-function. Furthermore, we can apply (20) in (40) to obtain the lower-bound bit error probability denoted as $P_{e}^{\mathrm{LB}}$.

To obtain the numerical results with the closed-form expressions and Monte Carlo simulation, we assume a total transmission power (dBm) given by $P_{T}=\sum_{i=0}^{2N-1}P_{x}\left[i,i\right]$, and $L_{\mathrm{CP}}\in \left\{14,18,30\right\}$ for representing the CP length values, and Δ∈{0,3,7} for the STO. Moreover, the gap factor Y=0 dB for the data rate analysis and the square 16-quadrature amplitude modulation (QAM) constellation for the BEP analysis. As for the B-geometry of the DOST, we consider B=64. The main parameters are listed in Table 3. Finally, all of the following numerical results were obtained from a Python 3.9 script written by the authors.

### 5.1. Impact of STO on the Interference

This section analyzes how the increase of Δ may affect the interference that disturbs the data communication. To do so, we rely on the Frobenius norm of (10), which is a matrix that captures the interference introduced by the CP length violation and the STO in the mapped vector. The Frobenius norm is given by

(42) $$\left|\right|{\underline{\bar{H}}}_{-i}{\left|\right|}_{F}=\sqrt{\mathrm{Tr}\left\{{\underline{\bar{H}}}_{-i}{\underline{\bar{H}}}_{-i}^{†}\right\}}.$$

**Figure 7:**
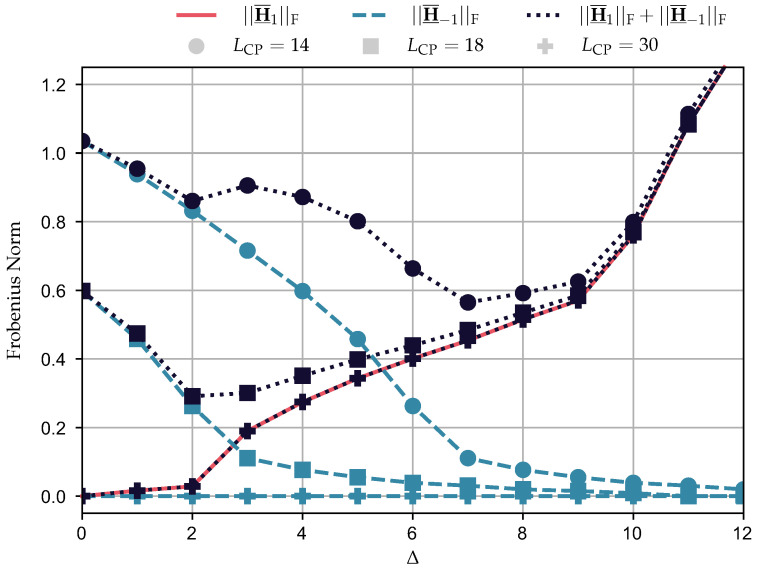
Frobenius norm of the matrices ${\underline{\bar{H}}}_{1},{\underline{\bar{H}}}_{-1}$, and ${\underline{\bar{H}}}_{1}+{\underline{\bar{H}}}_{-1}$ while considering Δ∈[0,12], $L_{\mathrm{CP}}\in \left\{14,18,30\right\}$.

As this equation informs the magnitude of ${\underline{\bar{H}}}_{-i}$, it can quantify the level of degradation yielded by the interference from the (l−i)th 2N-length symbol block into the *l*th 2N-length symbol block. Moreover, we consider h to be normalized (i.e., ${\left||h|\right|}^{2}=1$) because it facilitates the visualization of how the Frobenius norm of ${\underline{\bar{H}}}_{-i}$ behaves. Figure 7 shows the curves of $\left|\right|{\underline{\bar{H}}}_{-1}{\left|\right|}_{F}$, $\left|\right|{\underline{\bar{H}}}_{1}{\left|\right|}_{F}$, and $\left|\right|{\underline{\bar{H}}}_{-1}{\left|\right|}_{F}+\left|\right|{\underline{\bar{H}}}_{1}{\left|\right|}_{F}$ for Δ∈[0,12], $L_{\mathrm{CP}}\in \left\{14,18,30\right\}$. For all curves of $\left|\right|{\underline{\bar{H}}}_{-1}{\left|\right|}_{F}$ (i.e., all values of the CP), as expected, there is a decrease of its value with the increase of Δ until it reaches a value of zero. For instance, $\left|\right|{\underline{\bar{H}}}_{-1}{\left|\right|}_{F}=0$ when Δ=11 and $L_{\mathrm{CP}}=18$, which follows Theorem 1. However, the increase of Δ also increases $\left|\right|{\underline{\bar{H}}}_{1}{\left|\right|}_{F}$ in the same manner for all $L_{\mathrm{CP}}$ since it does not affect ${\underline{\bar{H}}}_{1}$. To conclude, the most interesting result is to analyze the sum of $\left|\right|{\underline{\bar{H}}}_{1}{\left|\right|}_{F}+\left|\right|{\underline{\bar{H}}}_{-1}{\left|\right|}_{F}$. Indeed, this sum shows that the choice of Δ can be used to mitigate the interference caused by the CP length violation in spite of the one originating from STO. In this context, Δ equal to 0, 2, and 7 results in the minimum values of $\left|\right|{\underline{\bar{H}}}_{1}{\left|\right|}_{F}+\left|\right|{\underline{\bar{H}}}_{-1}{\left|\right|}_{F}$ with an $L_{\mathrm{CP}}$ of 30, 18, and 14, respectively, yielding Frobenius norms of 0, 0.29, and 0.56. Section 5.4 and Section 5.5 discuss how this insight can benefit the achievable data rate and BEP, respectively.

### 5.2. Closed-Form Expressions and Monte Carlo Simulation Comparison

This section compares the results obtained with the closed-form expressions and the Monte Carlo simulation. The numerical results related to the closed-form expressions are due to obvious reasons reported based on $P_{e}\times P_{T}$ (dBm) plots. In addition, the Monte Carlo simulations are reported in terms of $\mathrm{BER}\times P_{T}$ (dBm) plots. As the lack of CSI at the transmitter is assumed, we apply UA together with adaptive modulation, meaning that all data communication schemes allocate the same amount of power and bits. Moreover, the adaptive modulation configuration is set only to perform the squared 16-QAM constellation, the frequency domain equalizer C-ZF is adopted, $L_{\mathrm{CP}}=14$, Δ=7, $P_{T}\in \left[-10,40\right]$ (dBm), and the PLC channel is corrupted by AWGN. To perform the Monte Carlo simulation, we assumed the transmission of $10^{7}$-equiprobable 2N-length symbol blocks, which are constituted by the points of the square 16-QAM constellation, resulting in the transmission of $1.024\times 10^{10}$ bits.

**Figure 8:**
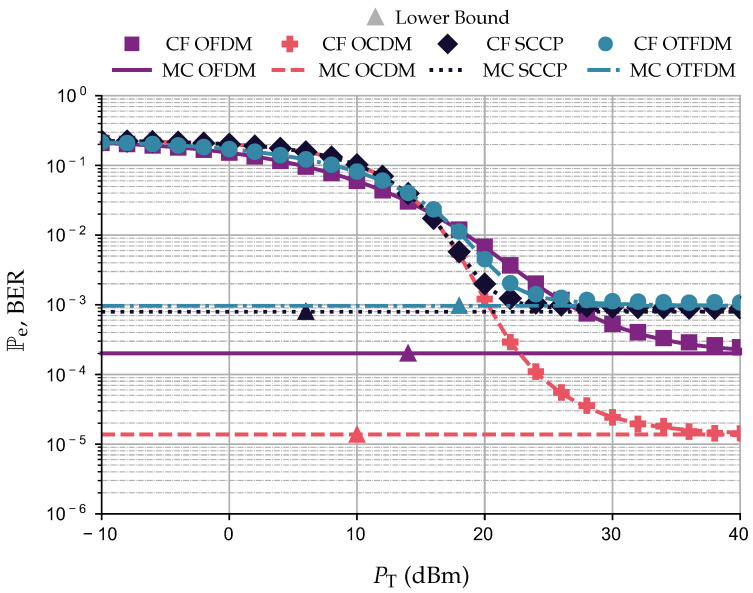
Comparison between closed-form expressions of $P_{e}$ and Monte Carlo (MC) simulations, i.e, BER, and $P_{e}^{\mathrm{LB}}$ for the OCDM and OTFDM while considering $L_{\mathrm{CP}}=14$, Δ=7, 16−QAM, and AWGN.

Figure 8 shows the numerical results for this comparison. There is a perfect agreement between the numerical results attained by the closed-form expressions (i.e., the $P_{e}$ curve) and Monte Carlo simulation (i.e., the BER curve). Moreover, after a certain value of $P_{T}$, its increase does not manage to improve the values of $P_{e}$ and BER. This behavior is expected since we consider the presence of the CP length violation and the STO. As a result, the SINR will reach the $P_{e}^{\mathrm{LB}}$ as $P_{T}$ increases; see (20). Consequently, both $P_{e}$ and BEP will reach a lower bound, as illustrated in Figure 8 by the horizontal lines. Since the numerical results obtained with the closed-form expressions and Monte Carlo simulation are in agreement, the following sections present only the numerical results obtained using the closed-form expressions.

### 5.3. Frequency Domain Equalization Comparison

This subsection discusses a numerical comparison between a few frequency domain equalizers (8). To perform this comparison, we adopted $P_{T}\in \left[-10,40\right]$ (dBm), the PLC channel corrupted by the presence of AWGN and ACGN, $L_{\mathrm{CP}}=14$, and Δ=7. Moreover, the UA technique is combined with the square 16-QAM constellation.

**Figure 9:**
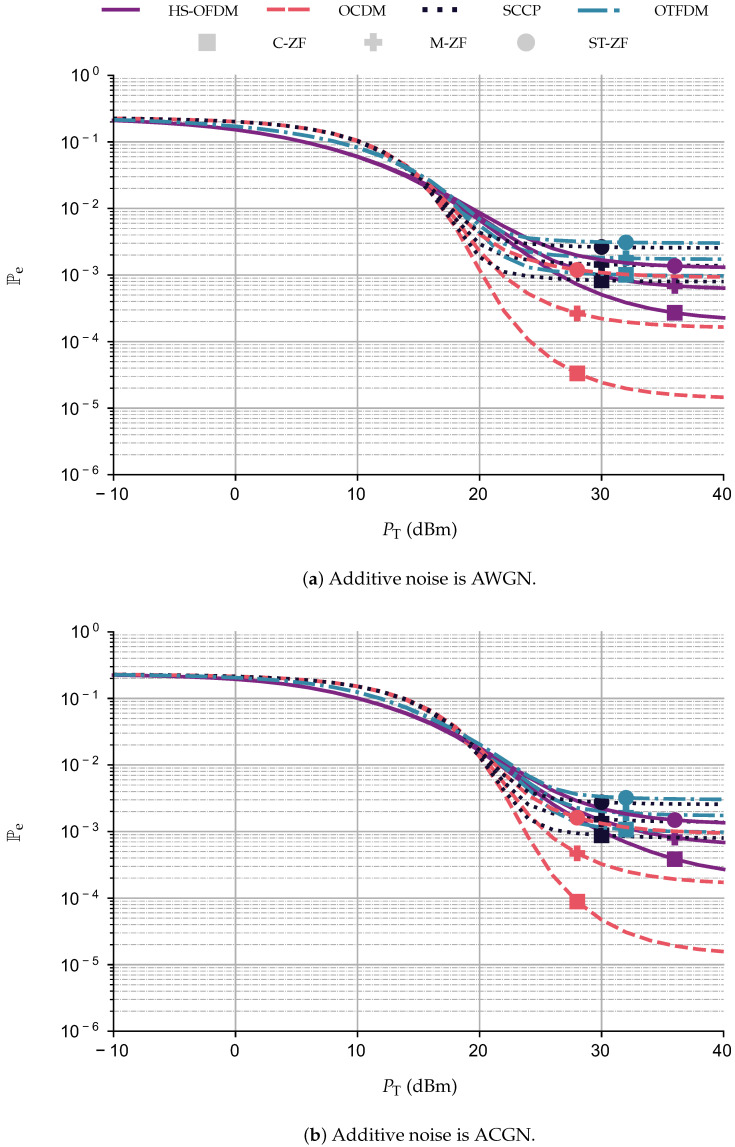
Comparison between the frequency domain equalizers C-ZF, M-ZF, and ST-ZF in terms of $P_{e}\times \;P_{T}$ (dBm) for the OCDM, HS-OFDM, SCCP, and OTFDM when $L_{\mathrm{CP}}=14$, Δ=7, square 16−QAM.

Figure 9 depicts the results attained with C-ZF, M-ZF, and ST-ZF. The performance curves show that the C-ZF scheme yields the best results, which is similar to what was reported in [27] for the OFDM scheme. For instance, while considering AWGN and $P_{T}=30$ dBm, HS-OFDM reaches values of $P_{e}$ equal to $5.06\times 10^{-4}$, $9.57\times 10^{-4}$, and $1.68\times 10^{-3}$ when considering C-ZF, M-ZF, and ST-ZF, respectively. Similar results were found using the other multiplexing modulation schemes. Under the same type of additive noise, the OCDM scheme obtains values of $P_{e}$ equal to $2.42\times 10^{-5}$ for the C-ZF scheme, while the other frequency domain equalizers, M-ZF and ST-ZF, yield $2.21\times 10^{-4}$ and $1.08\times 10^{-3}$, respectively. Moreover, for the SCCP scheme, considering the C-ZF scheme, the scheme reaches $P_{e}=8.28\times 10^{-4}$, while the other frequency domain equalizers obtained higher values of $P_{e}$. Moreover, the OTFDM scheme achieves a value of $P_{e}$ equal to $10^{-3}$ for the C-ZF scheme, $1.82\times 10^{-3}$ for the M-ZF, and $3.13\times 10^{-3}$ for the ST-ZF. Finally, similar behavior is also observed when AWGN is replaced by ACGN, but with higher values of $P_{e}$. All further numerical analyses regarding the data rate and BEP in the following sections use the C-ZF scheme since it yields the best results among all of the frequency domain equalizers considered.

### 5.4. Achievable Data Rate Comparison

This section discusses achievable data rates when UA and OA are considered. For the simulations based on UA, $P_{T}\in \left[-20,40\right]$ (dBm) is equally divided among the subcarriers, subchirps, subslots, and subtiles. On the other hand, the simulations using OA consider that the transmitter has complete knowledge of CSI to perform the water-filling algorithm to allocate $P_{T}\in \left[-20,40\right]$ (dBm) among the subcarriers, subchirps, subslots, and subtiles. Moreover, we consider $L_{\mathrm{CP}}\in \left\{14,18,30\right\}$ and Δ∈{0,3,7}. Regardless of the resource allocation technique (i.e., UA and OA), the nSNR value for OCDM and SCCP are the same for all subchirps and subslots, meaning that the total transmission power is equally distributed among the subchirps and subslots.

**Figure 10:**
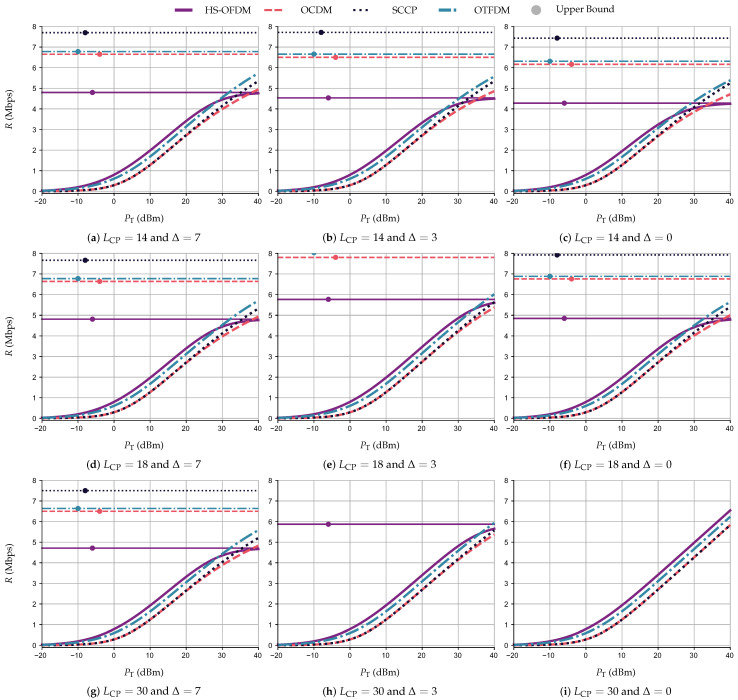
*R* (Mbps) $\times P_{T}$ (dBm) and $R^{\mathrm{UB}}$ for the HS-OFDM scheme, OCDM, and SCCP when $L_{\mathrm{CP}}\in \left\{14,18,30\right\}$, Δ∈{0,3,7}, and the additive noise is AWGN.

The numerical results in terms of *R* (Mbps) $\times \;P_{T}$ (dBm), while adopting UA and AWGN, are illustrated in Figure 10, with the constant lines being the achievable upper bound data rate, $R^{\mathrm{UB}}$. Note that Figure 10 depicts the results of the simulations performed while varying $L_{\mathrm{CP}}$ and Δ, with each row of subfigures having a fixed $L_{\mathrm{CP}}$ and each column a fixed Δ. Moreover, there is a Δ returning the best achievable data rate for each $L_{\mathrm{CP}}$, which agrees with the results discussed in Section 5.1. For instance, the use of Δ equal to 7, 3, and 0 results in the best curves for all schemes when $L_{\mathrm{CP}}$ is equal to 14, 18, and 30, respectively (i.e., the diagonal subfigures).

Indeed, varying Δ for each value of $L_{\mathrm{CP}}<L_{h}-1$, we can see that Δ=0 might not be the best choice for symbol synchronization under the interference caused by the CP length violation. This can be noticed by taking the $R^{\mathrm{UB}}$ as a parameter of the comparison. For example, with $L_{\mathrm{CP}}=18$, the $R^{\mathrm{UB}}$ for the OTFDM scheme is equal to 6.88, 8.03, and 6.77 Mbps with Δ equal to 0, 3, and 7, respectively. For the simulations without any form of interference, Figure 10i shows that the HS-OFDM scheme yields the best *R* for all values of $P_{T}$, reaching a value of 6.54 Mbps with $P_{T}=40$ dBm, while OCDM, SCCP, and OTFDM reach 5.83, 5.83, and 6.23 Mbps, respectively. However, with a growing interference, HS-OFDM experiences a greater loss of the achievable data rate. Indeed, HS-OFDM attains the lowest $R^{\mathrm{UB}}$ in the simulations with $L_{\mathrm{CP}}\neq 30$ and Δ∈{0,3,7}, while SCCP, OTFDM, and OCDM have the first-, second-, and third-highest $R^{\mathrm{UB}}$. Even though SCCP presents a higher $R^{\mathrm{UB}}$, OTFDM yields a better data rate than the other schemes when $P_{T}=40$ dBm, $L_{\mathrm{CP}}\neq 30$, and Δ∈{0,3,7}. For example, with $L_{\mathrm{CP}}=18$, Δ=7, and $P_{T}=40$ dBm OTFDM yields R=5.70 Mbps while HS-OFDM, OCDM, and SCCP reach *R* equal to 4.75, 4.93, and 5.32 Mbps, respectively.

**Figure 11:**
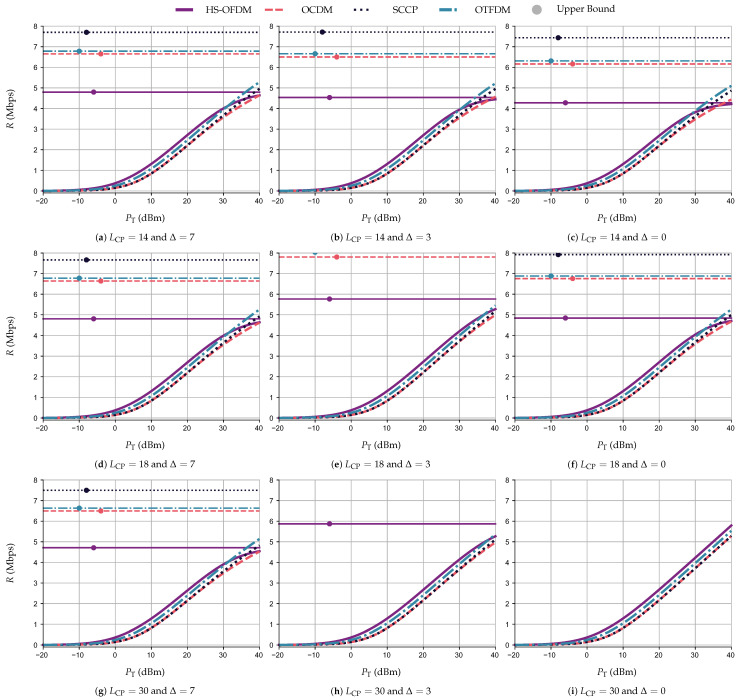
*R* Mbps) $\times P_{T}$ (dBm) and $R^{\mathrm{UB}}$ for the HS-OFDM scheme, OCDM, and SCCP when $L_{\mathrm{CP}}\in \left\{14,18,30\right\}$, Δ∈{0,3,7}, and the additive noise is ACGN.

Figure 11 displays the numerical results pertaining to UA and ACGN following the same structure of subfigures as Figure 10. Based on the curves, we can state that the analysis associated with UA and AWGN also applied to UA and ACGN. Indeed, without interference, the HS-OFDM scheme still attains the best achievable data rate. With interference, the SCCP scheme has the higher $R^{\mathrm{UB}}$ and the OTFDM scheme has the best data rate for $P_{T}=40$ dBm. For example, with $L_{\mathrm{CP}}=0$ and Δ=0, HS-OFDM, OCDM, SCCP, and OTFDM generate *R* equal to 5.79, 5.28, 5.28, and 5.53 Mbps, respectively, for $P_{T}=40$ dBm. Furthermore, with $L_{\mathrm{CP}}=18$ and Δ=7, HS-OFDM and OCDM yield R=4.64 Mbps, while SCCP and OTFDM generate *R* equal to 4.63 and 5.25 Mbps, respectively.

A comparison in terms of te achievable data rate for the two types of additive noise shows that with $P_{T}=30$ dBm, the HS-OFDM scheme yields a rate *R* equal to 4.97 and 4.22 Mbps for the AWGN and ACGN, respectively, while the OTFDM scheme provides the second best achievable data rate, producing values of *R* equal to 4.67 and 3.96 Mbps. Furthermore, the OCDM scheme and SCCP yield 4.26 Mbps and 3.71 Mbps, respectively, for the aforementioned additive noises. Moreover, even though the HS-OFDM scheme is more fragile to interference than the others, it yields better data rate results for most of the considered $P_{T}$ values in comparison to the other data communication schemes. Indeed, the HS-OFDM scheme might not be the best when $P_{T}$ is high and there is interference. This occurs when the interference compromises the data rate performance of the HS-OFDM scheme more than the others. For instance, with $L_{\mathrm{CP}}=14$ (i.e., a strong CP length violation), the HS-OFDM scheme still generates better achievable data rate results among the four data communication schemes for the values of $P_{T}\leq 25$ dBm for both additive-type noises. To put into numbers, when $P_{T}=20$ dBm, Δ=0, and AWGN, the HS-OFDM scheme yields a rate *R* close to 3.23 Mbps, the OCDM scheme attains R=2.67 Mpbs, the SCCP scheme yields 2.71 Mbps, and the OTFDM scheme attains R=3.07 Mbps.

**Figure 12:**
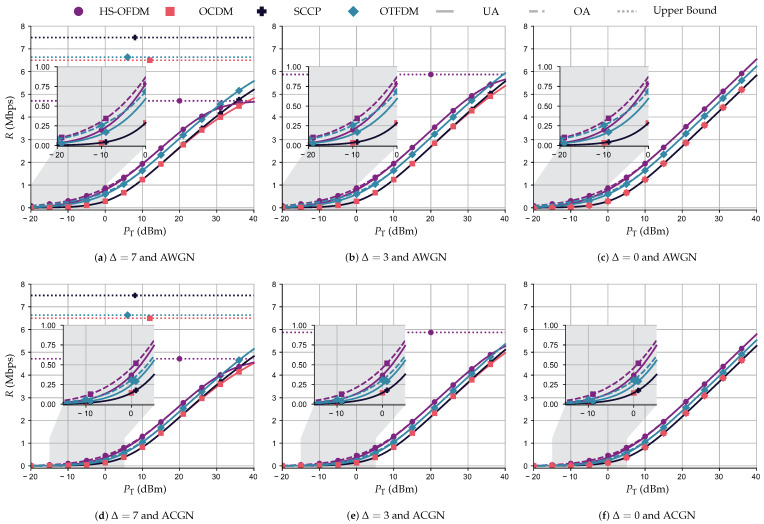
*R* (Mpbs) $\times P_{T}$ (dBm) and $R^{\mathrm{UB}}$ for the HS-OFDM scheme, OCDM, and SCCP schemes while considering $L_{\mathrm{CP}}=30$, Δ∈{0,3,7}, and both types of additive noise.

Performance comparisons in terms of *R* (Mbps) $\times \;P_{T}$ (dBm) while considering OA, through the water-filling technique, are in the plots shown in Figure 12. The nSNRs used to perform the power allocation is in Table 1 since we assume the transmitter does not know the existence of interference, but it knows PLC CIR and the additive noise. The knowledge of PLC CIR allows the receiver to use the correct value of $L_{\mathrm{CP}}$. In other words, the system does not face interference caused by CP length violation. The plots show that the OCDM scheme and SCCP attain the same performance for both resource allocation techniques since their nSNR are equal. In contrast, the HS-OFDM scheme and OTFDM with OA offer higher data rates than with UA when $-20\leq P_{T}\leq 0$ dBm for AWGN and $-15\leq P_{T}\leq 5$ dBm for ACGN. For example, while considering ACGN and Δ=7, the HS-OFDM scheme reaches a value of R=0.27 Mbps when adopting $P_{T}$ equal to −4 and −1 dBm with OA and UA, respectively. On the other hand, the OTFDM scheme requires greater values of $P_{T}$ than the HS-OFDM scheme to achieve the same *R*, reaching a data rate equal to 0.27 Mbps when $P_{T}=0$ dBm with OA and $P_{T}=2$ dBm with UA. Furthermore, the HS-OFDM scheme attains the highest achievable data rate for all values of $P_{T}$ in most scenarios (with and without interference). The exception is for high values of total power transmission ($P_{T}\geq 25$ dBm for AWGN and $P_{T}\geq 30$ dBm for ACGN), where the interference starts to harm the HS-OFDM scheme more than the other multiplexing techniques.

### 5.5. Bit Error Probability Comparison

This section focuses on the performance analysis in terms of BEP when the square 16-QAM constellation is used to transmit data (adaptive modulation). In other words, the CSI is not available at the transmitter side, and, as a consequence, subcarriers, subchirps, subslots, and subtiles transmit the same integer number of bits, which is equal to $b=\log_{2}16=4$. Note that this performance analysis is different from the one based on the use of UA, since the latter allocates b∈R in accordance with the nSNR, which is different from the adaptive modulation, in which b∈Z.

**Figure 13:**
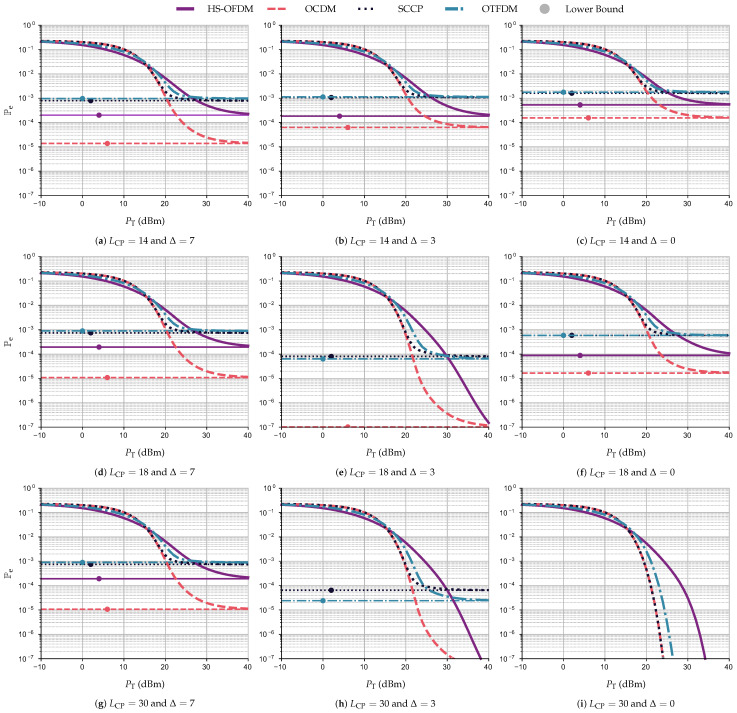
$P_{e}\times P_{T}$ (dBm) and $P_{e}^{\mathrm{LB}}$ for the HS-OFDM scheme, OCDM, and SCCP when $L_{\mathrm{CP}}\in \left\{14,18,30\right\}$, Δ∈{0,3,7}, and the additive noise is AWGN.

Figure 13 displays the graphs of $P_{e}\times P_{T}$ (dBm) while considering the PLC channel corrupted by AWGN, in which the constant lines refer to the lower bound BEP, $P_{e}^{\mathrm{LB}}$ for each data communication scheme. Note that, in this figure, each row of subfigures has a different $L_{\mathrm{CP}}$ from the others, and each column has a different Δ; moreover, the results are in accordance with Section 5.1. Meaning that, regardless of the scheme, each row of subfigures has a Δ that yields the lowest values of $P_{e}$. For instance, for the row with $L_{\mathrm{CP}}=14$, the $P_{e}^{\mathrm{LB}}$ for the OCDM scheme is equal to $1.54\times 10^{-4}$, $6.63\times 10^{-5}$, and $1.37\times 10^{-5}$ when Δ is equal to 0, 3, and 7, respectively, showing that STO can be helpful to mitigate interference caused by a CP length violation.

Regarding the performance of each scheme, when considering the scenario free of interference, the OCDM scheme and SCCP yield the best results, requiring only require $P_{T}=23$ dBm to achieve $P_{e}=10^{-6}$, while the HS-OFDM scheme and OTFDM require $P_{T}$ equal to 33 and 25 dBm, respectively. Moreover, as the interference starts to rise, the performance of all data communication schemes decreases. For example, for the simulations with $L_{\mathrm{CP}}=18$ and AWGN, to achieve a $P_{e}=10^{-4}$, the HS-OFDM scheme requires values of $P_{T}$ equal to 40 and 30 for Δ equal to 0 and 3, respectively. For the OCDM scheme, we can see that $P_{e}=10^{-4}$ when $P_{T}$ is equal to 23, 21, and 23 dBm for Δ equal to 0, 3, and 7, respectively.

Furthermore, for $L_{\mathrm{CP}}=14$, the OCDM scheme continues to yield better values of $P_{e}$ compared to the other data communication schemes. Moreover, the OCDM scheme is the only one to reach $P_{e}$ values lower than $2\times 10^{-4}$ for all Δ considered. In other words, the OCDM scheme offers a better (higher) performance loss in terms of $P_{e}$ than the others as the interference increases. In contrast, the SCCP scheme and OTFDM are proven to be the most fragile to interference in terms of $P_{e}$. Note that SCCP and OCDM share the same nSNR; however, the difference between their time–frequency domain occupation indicates that the former is less resilient to interference. This leads to the conclusion that the CSS-based data communication scheme is less impacted than the others regarding the interference caused by the CP length violation and the STO.

**Figure 14:**
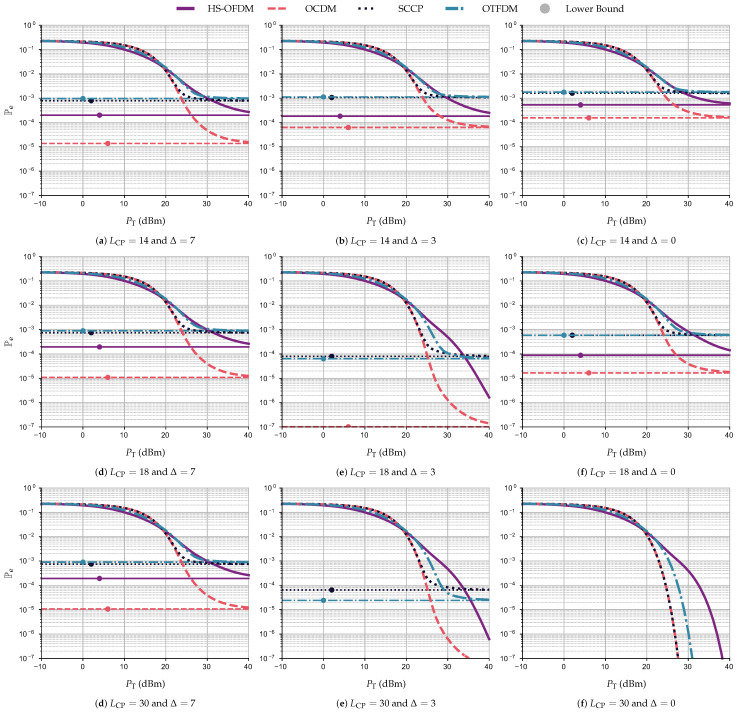
$P_{e}\times P_{T}$ (dBm) and $P_{e}^{\mathrm{LB}}$ for the HS-OFDM scheme, OCDM, and SCCP when $L_{\mathrm{CP}}\in \left\{14,18,30\right\}$, Δ∈{0,3,7}, and the additive noise is ACGN.

Moreover, Figure 14 shows the curves of $P_{e}\times P_{T}$ (dBm) and $P_{e}^{\mathrm{LB}}$ while considering the system operating under ACGN and following the subfigure structure of Figure 13. For these numerical simulations considering ACGN, all data communication schemes behave similarly to the simulations with AWGN as $L_{\mathrm{CP}}$, Δ, and $P_{T}$ vary. However, all data communication schemes when operating over PLC channels corrupted by ACGN require greater total transmission power than when the PLC channel is corrupted by AWGN to yield the same $P_{e}$. For instance, with $L_{\mathrm{CP}}=18$ and Δ=3, to achieve $P_{e}=10^{-6}$, the OCDM scheme requires $P_{T}$ equal to 27 and 30 dBm for AWGN and ACGN, respectively. Meanwhile, the HS-OFDM scheme requires 37 and 40 dBm for AWGN and ACGN.

To summarize, the OCDM scheme displays the best results when there is no interference, requiring almost 10 dBm less of the total power transmission to reach $P_{e}=10^{-6}$ than the HS-OFDM scheme and 3 dBm less than the OTFDM scheme. Moreover, the SCCP scheme and OCDM attain the same curve of $P_{e}\times P_{T}$ (dBm) without interference; however, the performance of the former degrades significantly with increasing interference. Relying on the numerical results, we can assert that the OCDM scheme is the best choice. Indeed, it shows better performance in terms of $P_{e}\times P_{T}$ (dBm), even in the presence of interference due to the CP length violation and the STO. However, if the computational complexity is one of the biggest concerns and the interference is absent, the SCCP scheme is better suited.

## 6. Conclusions

This paper discusses the performance degradation caused by CP length violation and the STO in data communication systems that rely on transmitting blocks of symbols over narrowband PLC channels corrupted by white or colored Gaussian random processes. Additionally, it introduces a unified formulation from which HS-OFDM, OCDM, SCCP, and OTFDM schemes are derived, providing the opportunity to design future multi-scheme transceivers capable of dealing with distinct sets of constraints faced by data communication systems. The paper also deduces closed-form expressions for estimated signal, SINR, and upper bound SINR for OCDM, SCCP, and OTFDM, allowing comparisons between them and HS-OFDM when the CP length violation and the STO occur.

Numerical results show that the closed-form expressions correctly characterize the performance of these data communication schemes. They also demonstrate that frequency domain equalization with different capacities presents similar behavior in all data communication schemes. Moreover, they show that STO can benefit the performance of data communication systems when the CP length violation occurs. The analysis of achievable data rates demonstrates that HS-OFDM and OTFDM typically provide the highest data rates in various scenarios, with or without interference. However, according to the BEP analysis, OCDM yields the most favorable outcomes for both types of noise, followed closely by SCCP and OTFDM without interference. In the presence of interference, HS-OFDM is the second-best alternative.

## Figures and Tables

**Table 1:** Summary of the nSNR.

Data Communication Scheme	nSNR
HS-OFDM	$|\Lambda_{H}{\left[k,k\right]|}^{2}P_{n}^{-1}\left[k,k\right]$
OCDM	${\left(\frac{1}{2N}\sum_{i=0}^{2N-1}\gamma_{\mathrm{nSNR}}^{-1}\left[i\right]\right)}^{-1}$
SCCP	${\left(\frac{1}{2N}\sum_{i=0}^{2N-1}\gamma_{\mathrm{nSNR}}^{-1}\left[i\right]\right)}^{-1}$
OTFDM	${\left(\frac{1}{\beta_{k}}\sum_{i=u}^{U}\gamma_{\mathrm{nSNR}}^{-1}\left[i\right]\right)}^{-1}$

**Table 2:** Computational complexity.

Transmitter		
**Scheme**	×	+
HS-OFDM	$4N\log_{2}\left(2N\right)$	$6N\log_{2}\left(2N\right)$
OCDM	$8N\log_{2}\left(2N\right)+2N$	$12N\log_{2}\left(2N\right)$
SCCP	−	−
OTFDM	$4N\log_{2}\left(2N\right)+\sum_{i=0}^{\nu -1}2\beta_{\nu }\left[i\right]\log_{2}\left(\beta_{\nu }\left[i\right]\right)+2N$	$6N\log_{2}\left(2N\right)+\sum_{i=0}^{\nu -1}3\beta_{\nu }\left[i\right]\log_{2}\left(\beta_{\nu }\left[i\right]\right)$
**Receiver**		
**Scheme**	×	+
HS-OFDM	$4N\log_{2}\left(2N\right)+E_{\times }$	$6N\log_{2}\left(2N\right)+E_{+}$
OCDM	$8N\log_{2}\left(2N\right)+2N+E_{\times }$	$12N\log_{2}\left(2N\right)+E_{+}$
SCCP	$8N\log_{2}\left(2N\right)+E_{\times }$	$12N\log_{2}\left(2N\right)+E_{+}$
OTFDM	$4N\log_{2}\left(2N\right)+\sum_{i=0}^{\nu -1}2\beta_{\nu }\left[i\right]\log_{2}\left(\beta_{\nu }\left[i\right]\right)+2N+E_{\times }$	$6N\log_{2}\left(2N\right)+\sum_{i=0}^{\nu -1}3\beta_{\nu }\left[i\right]\log_{2}\left(\beta_{\nu }\left[i\right]\right)+E_{+}$

**Table 3:** The main simulation parameters.

Parameters	Value
*N*	256
*B*	500 kHz
$L_{h}$	30
$L_{\mathrm{CP}}$	14,18,30
Δ	0,3,7
Y	0 dB
B	64

## Abbreviations

The following abbreviations are used in this manuscript:

PHY	Physical
HS-OFDM	Hermitian symmetric orthogonal frequency division multiplexing
OFDM	Orthogonal frequency division multiplexing
PLC	Power line communication
BER	Bit error rate
SCCP	Single carrier cyclic prefix
nSNR	Normalized signal-to-noise ratio
OCDM	Orthogonal chirp division multiplexing
CSS	Chirp spread spectrum
DFnT	Discrete Fresnel transform
OTFDM	Orthogonal time–frequency division multiplexing
DOST	Discrete orthogonal Stockwell transform
CP	Cyclic prefix
STO	Symbol timing offset
CFR	Channel frequency response
SINR	Signal-to-interference-plus-noise ratio
C-ZF	Complete zero-forcing
M-ZF	Modified zero-forcing
ST-ZF	Single-tap zero-forcing
UA	Uniform power allocation
OA	Optimal power allocation
BEP	Bit error probability
LTI	Linear time-invariant
CIR	Channel impulse response
WSS	Wide sense stationary
ISI	Inter-symbol interference
CSI	Channel state information
PSD	Power spectral density
IDFT	Inverse discrete Fourier transform
ICI	Inter-carrier interference
ICpT	Inter-chirp interference
IStI	Inter-slot interference
ITI	Inter-tile interference
AWGN	Additive white Gaussian noise
ACGN	Additive colored Gaussian noise
QAM	Quadrature amplitude modulation
